# The Role of Sister Cities’ Staff Exchanges in Developing “Learning Cities”: Exploring Necessary and Sufficient Conditions in Social Capital Development Utilizing Proportional Odds Modeling

**DOI:** 10.3390/ijerph120707133

**Published:** 2015-06-24

**Authors:** Patrick Henry Buckley, Akio Takahashi, Amy Anderson

**Affiliations:** 1 Huxley Environmental College, Western Washington University, Bellingham, WA 98225, USA; 2 School of Commerce, Meiji University, Tokyo 101-8301, Japan; E-Mail: takahasi@meiji.ac.jp; 3 Department of Mathematics, Western Washington University, Bellingham, WA 98225, USA; E-Mail: amy.anderson@wwu.edu

**Keywords:** sister cities, learning cities, social capital, proportional odds modeling, quality of life, sustainability, Japan

## Abstract

In the last half century former international adversaries have become cooperators through networking and knowledge sharing for decision making aimed at improving quality of life and sustainability; nowhere has this been more striking then at the urban level where such activity is seen as a key component in building “learning cities” through the development of social capital. Although mega-cities have been leaders in such efforts, mid-sized cities with lesser resource endowments have striven to follow by focusing on more frugal sister city type exchanges. The underlying thesis of our research is that great value can be derived from city-to-city exchanges through social capital development. However, such a study must differentiate between necessary and sufficient conditions. Past studies assumed necessary conditions were met and immediately jumped to demonstrating the existence of structural relationships by measuring networking while further assuming that the existence of such demonstrated a parallel development of cognitive social capital. Our research addresses this lacuna by stepping back and critically examining these assumptions. To accomplish this goal we use a Proportional Odds Modeling with a Cumulative Logit Link approach to demonstrate the existence of a common latent structure, hence asserting that necessary conditions are met.

“The Sister Cities Program is an important resource to the negotiations of governments in letting the people themselves give expression of their common desire for friendship, goodwill and cooperation for a better world for all.”

—President Dwight D. Eisenhower

## 1. Introduction

### 1.1. From Conflict to Cooperation

Much of the 20th century was one of increasing international tensions and conflict involving hot and cold wars, with urban areas dutifully fulfilling their role as pawns in the hands of the great powers that ruled them. However, first within the Western alliance after World War II and then later with the end of the cold war urban adversaries became cooperators, bridge builders, and eventually knowledge creators through social capital development. The Franco-German and American-Japanese relationships were prime examples with the free exchange of not only goods but also people and ideas through sister cities relationships [1,2,3,4]. Then with the collapse of the Soviet Union ending the Cold War cities throughout the developed world have begun to face new challenges raised by accelerating globalization and less national level support and control. Thus, cities strive for sustainability, environmental integrity, and maintenance of a high Quality of Life (QOL) in a world where they have been somewhat cast adrift from their old nation-state moorings [5,6]. This is affecting how cities interact horizontally and vertically both within their countries and internationally. At the same time, these places are being impacted by a myriad of ever changing factors such as shifting economic opportunities, aging populations, and changing ethnic makeup, creating both challenges and opportunities. Larger cities have responded to these changes with increased and targeted global cooperative networks and city-to-city staff and expert exchanges [7,8,9,10,11]. For small to mid-sized cities of the developed north (those under 100,000) whose needs are no less, but whose individual absolute resources are more modest, limited study has occurred, especially when it comes to understanding the value generated by city-to-city partnerships [12] particularly those from sister city type relationships (SCTR) [13]. Thus, an understanding of the value of small city SCTRs is important because “…it is one thing to be connected and another to derive value through city networks to help create and maintain sustainable cities” [7] (p. 170).

This study explores one such exchange—annual short term public sector staff exchanges between two Sister Cities: Bellingham, WA, USA and Tateyama, Chiba, Japan—using proportional odds modeling with a cumulative logit link to evaluate the potential for benefit flows resulting from increases of social capital created by the exchanges. Following Blanco and Campbell’s lead, it is argued that the staff exchanges positively affect the development of structural and cognitive social capital in each place which can be useful in decision making, local resource allocation, pursuit of sustainability, and ultimately local QOL. Which raises an important issue, what exactly is QOL, how is it measured, and how does it fit into this investigation?

Costanza *et al.* [14] define QOL and its measurement as:

*Quality of Life is the extent to which objective human needs are fulfilled in relation to personal or group perceptions of subjective well-being (SWB). Human needs are basic needs for subsistence, reproduction, security, affection, etc. SWB is assessed by individuals’ or groups’ responses to questions about happiness, life satisfaction, utility, or welfare. The relation between specific human needs and perceived satisfaction with each of them can be affected by mental capacity, cultural context, information, education, temperament, and the like, often in quite complex ways. Moreover, the relation between the fulfillment of human needs and overall subjective well-being is affected by the (time-varying) weights individuals, groups, and cultures give to fulfilling each of the human needs relative to the others*.

Thus QOL at a given place and time is based on the perception of the degree to which human needs are met through objective physical and human structures. However, as is noted these perceptions are highly subjective and effected by a number of traits including but not limited to culture, education, and information. Thus, if two groups evaluate the same place, it is not guaranteed that they will come to the same conclusion. This becomes problematic if in fact the goal is an exchange of ideas/knowledge between the two groups to enhance QOL and consequently sustainability. What is required is a demonstration that distinctly different groups start from a similar framework and benchmark before one can expect the creation of a valuable knowledge exchange. In social capital terms this refers to the need for a common cognitive social capital framework before one can demonstrate the value of structural social capital. Exploring the staff exchanges for evidence of a common cognitive social capital framework through the use of QOL indicators is the focus of this study.

The remainder of this paper is divided into the following parts. This section continues with a review of the literature and defines terms. Next is a brief statement of the objective of this study—which focuses on demonstrating the existence of a common latent QOL framework upon which each city fits, a necessary initial condition in order for common benefits to be generatable from the SCTR staff exchanges. This is done through the comparison of subjective evaluation of QOL indicators by staff members from each city focused on their own city and the sister city. Given the size constraints of this paper, it should be noted that only necessary conditions, demonstration of cognitive social capital via QOL exploration, are presented here, a condition that if not met makes further study for sufficient conditions—benefit streams emanating from the exchanges via cognitive social capital—a moot point. The Introductory section concludes by providing background information on SCTRs in general and Bellingham and Tateyama in particular. Section 2 introduces the descriptive results of the data collected from the staff exchange participants focusing on the QOL measures themselves and a brief discussion of their summary results. Section 3 focuses on the results of the methodology applied to analyze the data for latent structure, proportional odds modeling with a cumulative logit link. Section 4 interprets the model’s results and explores its goodness of fit. Section 5 is a discussion of the particular results for our target cities and lays the ground work for moving to the next level of analysis, an exploration of meeting sufficient conditions. A short conclusion then ends the paper.

### 1.2. Learning Cities, Urban Neural Knowledge Networks, and Social Capital

A learning city [15,16] is a proactive innovator that relies on the continuous infusion, evaluation, sharing, and implementation of knowledge. What differentiates it from any networked urban place is that it goes beyond the traditional act of collection and archiving knowledge, the *hard infrastructure* of learning, to the more important development and encouragement of a culture of learning, sharing, disseminating, innovating, and strategically applying the knowledge the *soft infrastructure*. Basically it is “…a learning environment that is characterized by trustworthy relationships, a culture of sharing, and a willingness to collaborate” [16] (p. 12) both internally and externally, resulting in what is defined here as an *urban neural knowledge network* (UNKN) [17]. The resulting UNKN is based not simply on traditional institutional connections but also connections that arise in an *ad hoc* and opportunistic form of what Leresche and Saez [18] have termed a type of human *synapsis*. Such a synapsis is a connection that develops at a very fine level as individuals, organizations, and/or institutions seek out one another to address pressing issues of common interest and develop social capital. Issues addressed in this fashion are ones that cross traditional boundaries, thus ignoring formal geographic and institutional bounds hence allowing the problem to define the linkages in the network. Such connections can then be broadened and thickened as appropriate or wither and die as problems are successfully addressed, ignored, or other avenues pursued. Leresche and Saez describe such a flexible form of problem solving as an *adhocratic* form of governance which augments but does not replace the traditional centrally controlled hierarchical *topocratic* government. Campbell [19] has diagrammed such a network for the city of Torino, Italy. Similar types of *ad hoc* networking have come to typify problem solving across international boundaries in areas commonly defined as *cross-border regions* [20].

Large cities, those ranging from a half million upwards, have substantial public and private resources to develop and nurture UNKN’s and create local “innovative milieus” [21] fostering social capital development. Smaller sized cities, especially those under 100,000, face daunting resource constraints but are no less in need of forward looking activity to sustain their competitiveness and QOL. Unable to participate in large intensive international information networks, especially those created by mega cities [8], they must rely on more modest means. One resource that is available to urban areas of all sizes is a SCTR which provides low cost opportunities for exchanges, knowledge base building, and learning. As a result, it should be no surprise that participation by cities under 100,000 in absolute numbers dominates such relationships; they make up 58% of the partnerships compared to 42% by larger cities [22,23]. The question that remains unanswered is demonstrating the usefulness and value created by these relationships.

### 1.3. Study Objective

The underlying thesis of our research is that great potential value can be derived from international networking and city-to-city exchanges such as SCTRs through social capital development that results in greater urban sustainability as manifested in improved urban economies, environments, and over-all QOL. However, such study must differentiate between necessary and sufficient conditions for the key ingredient of social capital creation, as well as its separate parts, structural and cognitive factors. Past studies assumed necessary conditions were met and immediately jumped to demonstrating the existence of structural relationships by measuring networking while further assuming that the existence of such demonstrated a parallel existence of cognitive social capital development [7]. Our research program addresses this lacuna by stepping back and critically examining these assumptions, specifically this paper focuses on an investigation of necessary conditions, existence of a common structural framework into which social capital can be infused. Later work will investigate the infusion of cognitive social capital in conditions where networking exists. To accomplish this goal we use a Proportional Odds Modeling with a cumulative logit link approach to explore for a common latent structure, hence demonstrating that necessary conditions are met. To be clear, we hold that without a common underlying structure or framework which would provide the basis for parallel but not necessarily identical UNKNs and knowledge sharing and utilization, benefits from networking, especially from cognitive social capital generation, would be stunted at best and non-existent at worst.

### 1.4. Sister City Type Relationships

Current estimates suggest that between 15,000 and 20,000 SCTRs exist worldwide (UNDP 2000). The objectives of these people to people para-diplomatic activities has evolved and changed over time [24], but all can be summarized under three general goals: (1) building mutual global cooperation from the municipal level, (2) promoting friendship and cultural understanding, and (3) stimulating sustainable development [22], or simply cities learning and gaining from one another under what Cremer, de Bruin, and Dupuis [25] have called a form of Municipal-Community Entrepreneurship [26].

These people to people relationships operate at two levels of interaction one at the personal micro level and second at the meso community level with a hoped for long-term overarching goal of macro level consequences. As noted, the objectives in pursuit of these goals have varied. Recently economic development and sustainability have moved to the fore [25]. Studies evaluating these relationships have focused primarily on north-south linkages at the meso level [27]. Investigation of symmetrical north-north relationships has been much more limited. Those that do exist tend to be part of larger scale national reviews which can include north-south relationships as well [25,28,29,30]. Thus, this study breaks new ground in two ways, first it focuses on a symmetrical north-north relationship and second it concentrates on the micro level, answering Zelinsky’s [4] call for analysis at the rank and file level.

### 1.5. Background: Bellingham and Tateyama

Bellingham and Tateyama began their relationship in 1958 as one of the earliest linkages between Japan and the United States, especially for small cities. In 1992, city hall staff exchanges were instituted with a purpose of “…review(ing) each other’s policies and procedures and the facilities available” [31] (p. 1). These exchanges were designed to be of one week in duration by two middle management staff members on an annual basis.

Given the broad mandate of these exchanges and their modest duration and resource demands could much be expected from these low-level fact finding missions? This seems to be exactly Zelinsky’s [4] question in asking whether such efforts cause attitudinal shifts among the rank and file and decrying the fact no in depth research has been performed.

## 2. Data Collection and Descriptive Results

Between 1990 and 2006 Bellingham sent 32 staffers and Tateyama 34, all were invited to participate in the study in early 2007 [32]. From this universe Bellingham provided 19 respondents a participation rate of 59% and Tateyama 27 or 79% participation. As noted earlier the full study had two purposes, first to determine if a common structural framework exists for the evaluation of current QOL what we refer to as necessary conditions which are reported on here, and second to determine if there was an infusion of cognitive social capital, hence the successful demonstration of meeting sufficient conditions, to be reported on in a latter paper. For the entire study a four round Delphi was conducted, of which only the first round data is utilized here. During the study all interviews were carried out following the rules of the Declaration of Helsinki of 1975 (http://www.wma.net/en/30publications/10policies/b3/), revised in 2008. Approval for the study instruments and was obtained from the Western Washington University Human Subjects Review Committee in June of 2006. Written informed consent was obtained from each participant prior to their inclusion in the study.

In the first round of the multi-round Delphi study [33], panelists were asked to evaluate and discuss 11 specific indicators of QOL for each city on a scale of 1 to a high of 1,000 as well as provide an over-all value of QOL in each city, item 12 (see Table 1 for a complete list of QOL indicators). The indicators were created based on Costanza *et al.*’s definition of QOL and grouped into three broad categories reflecting the physical, service, and social environment [34]. Average results by each panel for each city are provided in Table 1, along with measures of significant differences from the Wilcoxon signed rank sum test, and a summary symbol indicating which city scored higher when significant differences are found.

From the center columns of Table 1 it is clear that the Bellingham panel rates all the indicators and the summary measure of QOL for itself equal to or significantly [35] higher than Tateyama except for the measure of public safety, a not surprising result. The significantly higher values for housing, parks, leisure, education, and shopping may be attributed to basic differences in things like population density, Bellingham’s prime location for recreation between mountain and sea, the existence of a comprehensive university in Bellingham, and the city’s role as a major retail outlet for Canadian cross-border shoppers from Canada’s third largest metropolitan area. However, the interesting result is that the Bellingham panel ranks transportation equal in both places despite a considerable difference in infrastructure and modal choices. Likewise the Bellingham panel finds the social environment equal in both places.

The Tateyama panel results (right side of Table 1), collected completely independently, generally agree with the significantly higher values noted above with one exception, shopping, which they rank as equal between the two cities. However, by looking at the mean values it is clear that this is not a sign that the Tateyama panel thinks that Tateyama has better shopping opportunities then identified by the Bellingham panel, but rather that they may be less impressed by or knowledgeable of the opportunities in Bellingham. The most interesting thing from the Tateyama panel is they rank the quality of their transportation and social environment significantly lower than Bellingham’s.

**Table 1:** Average of QOL indicators by panel between cities and Wilcoxon Signed Ranked (WSR) Test results.

		Bellingham Panel				Tateyama Panel			
		Bellingham	Tateyama	WSR results	Summary^a^	Bellingham	Tateyama	WSR results	Summary^a^
*Group*	*Indicator*	*Mean*	*Mean*	*sig.*		*Mean*	*Mean*	*sig.*	
Physical	1. Quality of Housing	821	711	0.028	B**	794	576	0.000	B***
Physical	2. Quality of Transportation	708	781	0.188	---	719	520	0.000	B***
Physical	3. Parks and Outdoor Recreation Opportunities	909	687	0.001	B***	822	470	0.000	B***
Services	4. Opportunities for Leisure and Amusement	795	736	0.083	B*	800	502	0.000	B***
Services	5. Opportunities for Educational Enrichment	822	738	0.075	B*	736	563	0.002	B***
Services	6. Opportunities for Shopping	800	624	0.003	B***	679	643	0.191	--
Services	7. Feeling of Personal Safety and Security	721	861	0.007	T***	642	761	0.001	T***
Social	8. Environment for Families	808	787	0.330	---	743	670	0.025	B**
Social	9. Environment for Children	776	784	0.725	---	728	674	0.016	B**
Social	10. Environment for Retired People	761	716	0.609	---	723	663	0.053	B*
Social	11. Feeling of Community and Volunteer Spirit	746	707	0.929	---	775	604	0.001	B***
Over-all	Over-all Quality of Life	853	758	0.023	B*	767	650	0.001	B***

a. The summary measure combines the letter of the city with the higher mean with WSR test significance results: B* sig < 0.10, B** sig < 0.05, B*** sig < 0.01.

Looking at these same results in a slightly different fashion by directly comparing the two panels’ evaluations for each city (Table 2) and using the Wilcoxon-Mann-Whitney test produces a rather strong agreement concerning Bellingham but quite a bit of difference regarding Tateyama. In evaluating Bellingham only three indicators plus the summary QOL measure are significantly different with the Bellingham panel assigning each of these higher numbers; these are Parks, Shopping, and Safety. Given the fact that Bellingham ranks near the top in park acreage per capita for Washington State, it is not surprising that the Bellingham panel would provide a higher value than Tateyama visitors. Likewise we have already discussed why shopping values might be different. Finally, in regards to safety the higher value from the Bellingham panel might indicate a different relative perspective on what is acceptable from an American viewpoint, thus celebrating the relative safety of Bellingham.

**Table 2:** Average of QOL indicators for each city by different panels and Wilcoxon-Mann-Whitney (W-M-W) Test results.

		Bellingham Values				Tateyama Values			
		*Response from*				*Response from*			
		Bellingham panel	Tateyama panel	W-M-W results	Summary^a^	Bellingham panel	Tateyama panel	W-M-W results	Summary^a^
*Group*	*Indicator*	*Mean*	*Mean*	*sig.*		*Mean*	*Mean*	*sig.*	
Physical	1. Quality of Housing	821	794	0.562	---	711	576	0.036	Bm**
Physical	2. Quality of Transportation	708	719	0.685	---	781	520	0	Bm***
Physical	3. Parks and Outdoor Recreation Opportunities	909	822	0.085	Bm*	687	470	0.001	Bm***
Services	4. Opportunities for Leisure and Amusement	795	800	0.718	---	736	502	0	Bm***
Services	5. Opportunities for Educational Enrichment	822	736	0.131	---	738	563	0.003	Bm***
Services	6. Opportunities for Shopping	800	679	0.009	Bm***	624	643	0.645	--
Services	7. Feeling of Personal Safety and Security	721	642	0.054	Bm*	861	761	0.028	Bm**
Social	8. Environment for Families	808	743	0.148	---	787	670	0.009	Bm***
Social	9. Environment for Children	776	728	0.434	---	784	674	0.035	Bm**
Social	10. Environment for Retired People	761	723	0.657	---	716	663	0.266	--
Social	11. Feeling of Community and Volunteer Spirit	746	775	0.551	---	707	604	0.067	Bm*
Over-all	Over-all Quality of Life	853	767	0.022	Bm**	758	650	0.005	Bm***

a. The summary measure combines letters for the city with the higher mean with the W-M-W test significance results: Bm* sig < 0.10, Bm** sig < 0.05, Bm*** sig < 0.01.

When turning to Tateyama, the Tateyama panel consistently ranks itself lower than the Bellingham panel on all but two measures, shopping (discussed previously) and environment for retirees. The equality of the retirees’ environment measure seems to be a result of the Bellingham panel ranking this measure quite low, considerably lower than the environment for family or children rather than the Tateyama panel scoring it high. Clearly the Tateyama panel is much more critical of or perhaps modest about itself then the visitors which is also affirmed in the summary over-all QOL measure.

Based on these descriptive results from each city a number of general conclusions can be drawn here: (1) Bellingham emerges from this set of indicators as generally equal to or superior to Tateyama except for public safety as scored by both panels and (2) the Bellingham panel tends to assign higher scores although not always significantly higher, but this is most notable when the two panels evaluated Tateyama. This last point might be an indicator of cultural bias which has been seen in other studies using Likert like scales [36]. Although this descriptive analysis provides insight into individual similarities and differences, it only does so in a bivariate fashion. Our next task is to apply a multivariate technique to this data to discover if it represents random and unconnected variations or a systematic yet different positions on the same QOL framework.

## 3. Methodology: Proportional Odds Modeling with a Cumulative Logit Link

To search for a common latent structure, a type of ordinal regression rather than linear regression was chosen. Proportional odds modeling with a cumulative logit link was used to model the ratings of our pair of sister cities on twelve QOL indicators. Forms of proportional odds modeling has been widely used in QOL studies especially in the medical field [37,38], but less so in looking at urban QOL issues [39,40]. However, the focus of such earlier work has been different; it has been based on creating explanatory models where a series of independent variables are used to explain ordinal dependent QOL measures. Here the focus is not to explain but to compare the results from two groups of respondents to look for the existence of a common latent QOL structure identifiable to and used by both groups. As noted above, there were twelve QOL questions, each of which was asked twice: once about Tateyama and once about Bellingham. Individuals were asked to respond to each question with a value between 0 and 1000. The great majority of individuals gave responses of 100, 200, 300, . . . , 1000, as if this were an ordinal response variable with 10 or fewer categories. As a result, we decided that it would be most appropriate to treat the data as ordinal responses and choose ordinal regression over the more common linear regression [41]. Second, given the scarcity of data below a value of 500, and very few values that did not fall exactly on a hundred entry, we decided to reduce the number of categories. We binned the responses as follows: a raw score between 0 and 500 was coded as rank 1, the lowest; a score in the 501–600 range was coded as a 2, a score between 601 and 700 was coded as a 3, and so forth, with a score between 901 and 1000 coded as a 6. This gave a total of 242 1’s, 115 2’s, 164 3’s, 270 4’s, 187 5’s, 90 6’s, and 36 missing responses. We felt this binning scheme that collapsed values of 0 to 500 into a single bin and all others into their own based on steps of one hundred accurately reflected the over-all response pattern [42]. See Equation (1): Two city QOL proportional odds model with a cumulative logit link:

Logit [P(Yikm ≤ j)] = θj − βk − γkWm − α1Xi − α2Wl − δXiWm – ui
(1)

In applying our model (Equation (1)) to test if individuals from Tateyama and Bellingham gave the same or systematically different responses to the QOL questions the following variables were included in the model: (1) the respondent’s city (Xi, which is 0 if individual i is from Tateyama and 1 otherwise), (2) the city being asked about (Wm, which is 0 if city m is Tateyama and 1 otherwise), and (3) which of the 12 questions was being asked (to limit clutter and as is a common way of presenting such a model, in equation 1 we provide no specific symbol for this variable, instead the equation itself is written for a specific k or question, meaning that a vector of twelve of these equations are utilized), and (4) the dependent QOL indicator’s ordinal score (Yikm, which refers to the rank score given by individual i for question k about city m). (5) We also included an interaction term between the question being asked and the city the question was asked about (Xi Wm, which equals 0 except for a case of individual i coming from Bellingham and evaluating city m, Bellingham, where it equals 1). Finally, (6) to account for correlation between responses from a single individual, we included a random effect for each individual. This random effect, ui, was assumed to follow a normal distribution with a mean of 0 and a variance σ^2^. In this way, we were able to look at the twelve quality of life questions in aggregate and draw conclusions about overall patterns [43].

Thus, in our model, the log-odds that individual i will give a response less than or equal to rank j when asked question k (k = 1, 2, . . . , K) about city m is given by equation 1 above.

Solution of this proportional odds model requires relative calibration, in this case around one of the K questions. We selected Question 12, an estimate of overall QOL, as our baseline around which all other QOL measures are arranged. This results in β12 and γ12 being constrained to be zero. In addition, since it simultaneously fits logistic regressions for all choices of j (j = 1, 2, . . . , J − 1), no regression is fit for j = J (or j = 12 in our particular case) because P(Yikm ≤ J) = 1.

The model estimates six parameters. The first θj acts as a set of monotonically increasing intercepts or thresholds. As the sum of the ordinally ranked evaluations j increase from 1 through J-1, so too does θj. Next, βk captures any individual differences between QOL measure k (Question k) and the baseline measure, in our case the overall QOL measure from Question 12. Thus, the individual measure k can be equal to Q12 or significantly higher or lower than this baseline. On the other hand, γk captures individual differences in QOL measure k between Tateyama and Bellingham. While both cities are assumed to occupy positions on the same QOL framework, differences in resource endowments and policies will cause variations in position on the structure for different indicators. Then α1 captures a systematic shift in the ordinal response by panelists from one city *versus* the other, what we have assumed to be a cultural shift as discussed in the previous section. As noted, it has been well documented in the literature that culture can cause such a systematic shift in ordinal responses when evaluating the same entity. Next α2 captures a systematic across the board resource or policy shift (as opposed to a specific one captured by γk above) in QOL between two target cities. Basically this indicates that one city has a generally higher/lower QOL than the other, thus indicative of each city having different positions on the latent structure based on a systematically different set of resource endowments and/or policies. Finally, δ captures a relative interaction shift that results from a respondent evaluating their own city *versus* the other, potentially reporting a systematically higher or lower value than would otherwise be expected. It is conceptualized as capturing additional knowledge resulting from greater intimacy with one’s own area that can only be accomplished by an individual who has been integrally involved over the long term with a specific place, knowledge which is not duplicable during a short visit. Thus, it can only be applied by respondents to their own place of residence. Also, as a relative term it can be estimated by focusing either on Tateyama or Bellingham. Either way δ would produce the same numeric value but in each case respectively an opposite sign would appear.

Analysis was run using the Christensen’s ordinal package in R [44].

## 4. Discussion of Model Results

### 4.1. Interpreting the Model

**Figure 1:**
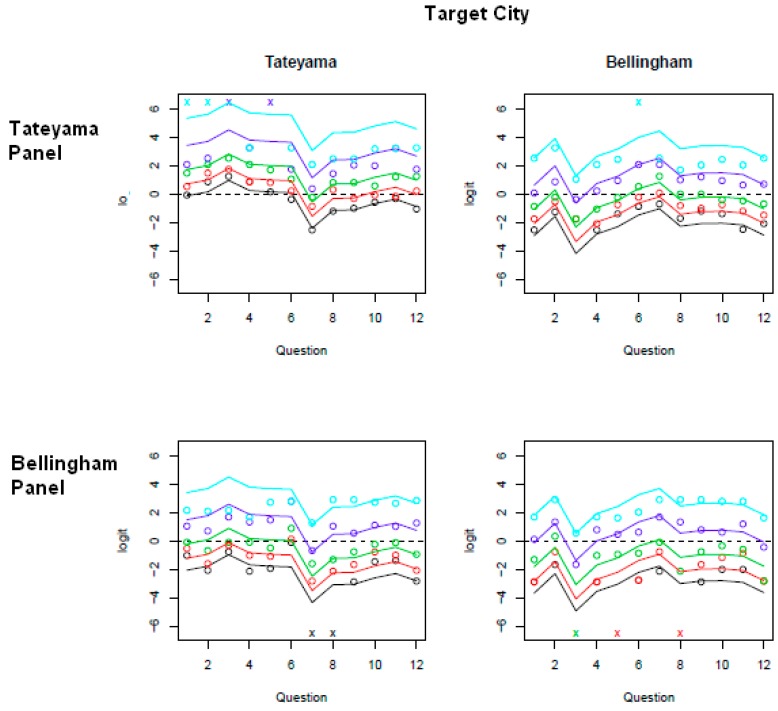
QOL Model fit to Sister-city QOL indicators. The top (bottom) row of plots give the fitted values when the respondent was from Tateyama (Bellingham). The left hand (right hand) column gives plots when the questions were asked about Tateyama (Bellingham).

Figure 1 shows the fit of our model to the data, with colors coding the various cumulative logits, namely logit (P(Y ≤ j)) for j = 1 (black) lowest rank for QOL measures, j = 2 (red), j = 3 (green), j = 4 (purple), and j = 5 (light blue) highest cumulative rank for QOL measure save for cumulative rank 6 which would include all respondents and reach the top of the diagram. To be clear as we move up the diagram, black to red to green… we are actually cumulating more and more of ever increasing QOL rankings until all rankings are accounted for at the top of the diagram. The gap between the top of the diagram and the light blue line indicates the remainder of our results, which are in the highest QOL rank j = 6. The larger this gap the greater the expected assignment of rank 6 in our data set for a particular QOL indicator with one caveat explained below.

Thus, the expected cumulative logit value based on the model, for individual i who responded with level j to question k whose random effect value is u = 0, is indicated by colored line j. The color coded circles represent the observed logit [45] values for actual j’s and k’s from the dataset with the following exceptions. If all individuals gave a rank of the number 4 or lower on a question, we would observe logit (P (Y ≤ 4)) = log[P (Y ≤ 4)/(1 − P (Y ≤ 4))] = log(1/0) = ∞ (indicating that there were no high or very high ranks for QOL recorded on this item). Essentially this indicates a truncated set of raw data across our entire 1 (lowest) to 6 (highest) range where some of the highest ranks of QOL were not assigned, 5 and 6 in this example. In this case, we marked a purple “x” at the top of the plot. In comparison, a light blue “x” on top indicates that all individuals assigned a rank of 5 or lower. At the opposite end of the range, if no individual gave a score below a particular value (consensus placed all responses above a given rank), we would get a logit value of −∞, and we denoted this with an color coded “x” at the bottom of the plot, where the color of the “x” indicates the lowest score such that all responses were above that value.

The plots (Figure 1) show the cumulative log-odds of giving a response at or below some rank. For example, in the top-left plot or northwest quadrant of the figure, the dip for question 7, about QOL of safety, indicates that Tateyama natives had a low log-odds of giving their city low scores for question 7. In other words, Tateyama natives tended to score their city highly for safety. This is also indicated by the large gap between the top of the diagram and light blue line for this question. Hence, lines congregated towards the bottom of a box in the figure correspond to questions that received a greater number of high QOL scores/ranks. In a similar fashion looking immediately below to the box in the southwest corner of the display, Bellingham panelists also indicated low log-odds scores for Tateyama regarding the safety measure. In addition, all of them chose a rank greater than j = 1. This resulted in the black “x” at the bottom of the box. Finally, again returning to the upper northwest quadrant it should be noted that question 1 and 2 have light blue “x’s” indicating that none of the panelists from Tateyama scored QOL of housing or transportation at the highest rank of 6 in their home city of Tateyama. Similarly, they also failed to score question 3 and 5, parks and educational measures, above the purple rank of 4.

Further aspects of the figure illustrate that the lines for the different logits are parallel—the vertical distance between any pair of lines (e.g., light blue and purple) is constant. Further, that same distance is maintained among all plots within the figure. This phenomenon is the result of the proportional odds assumption and is part of our model. On the other hand, the fact that the lines are not evenly spaced between different logits (e.g., the red and black lines are closer together than the light blue and purple lines) indicates that we made no restrictions on the θ values, which is further illustrated with the threshold values in Table 3.

**Table 3:** Threshold Parameter Values.

Parameter	Estimate	Std. Error	Z-value
**θ1**	−0.877	0.388	−2.261
**θ2**	−0.037	0.386	−0.096
**θ3**	0.975	0.388	2.511
**θ4**	2.674	0.395	6.763
**θ5**	4.587	0.415	11.05

Turning to the other parameters, first those focusing on the QOL indicator questions, which relate to parameters βk and γk (Table 4 and Table 5), the following results were generated. Given that Xi equals 0 for Tateyama, it is impacted only by βk values indicating that the first six of its eleven QOL measures (representing physical and service measures) are significantly lower than its overall QOL measure (the twelfth measure) and the seventh one (safety) is significantly above. This is also illustrated in Figure 1, in the two boxes describing Tateyama (contained in left column) the logit line for questions 1 through 6 is located at a higher level than that of question 12 and question 7 substantially below. Bellingham on the other hand is impacted by the additive combination of both parameters βk and γk. Here we will concentrate on where a combination of significant changes alters the above results or a new significant impact is present. On measure 3, parks and recreation, the much higher positive γ3 parameter reverses the impact from the negative β7 indicating that this indicator receives higher rankings then the over-all QOL measure. On measure 4 the combined results are near zero, with a very slight positive influence for leisure activities. However, for safety, question 7, the combined impacts reverse signs to a distinct negative change from the overall QOL measure. The only other changes for Bellingham vis-a-vis the overall QOL are slight negative impacts from questions 8 and 9 related to the environment for families and children. Again these results are easily illustrated in the right hand column of Figure 1 especially the very strong positive shift for parks and recreation, Q3, and negative reversal for safety, Q7.

**Table 4:** Parameters for variation of QOL from over-all QOL.

Parameter	Estimate	Std. Error	Z-value	*p*-value	Significance ^a^
**β_1_**	−0.751	0.400	−1.88	0.06036	*
**β_2_**	−1.031	0.410	−2.51	0.01204	**
**β_3_**	−1.842	0.420	−4.38	1.17E-05	***
**β_4_**	−1.131	0.401	−2.82	0.00479	***
**β_5_**	−1.032	0.403	−2.56	0.01043	**
**β_6_**	−0.977	0.407	−2.4	0.01637	**
**β_k7_**	1.525	0.384	3.97	7.21E-05	***
**β_k8_**	0.273	0.372	0.73	0.46283	
**β_k9_**	0.237	0.381	0.62	0.5331	
**β_10_**	−0.209	0.403	−0.52	0.60337	
**β_11_**	−0.514	0.402	−1.28	0.20101	

^a.^ * sig < 0.10, ** sig < 0.05, *** sig < 0.01.

**Table 5:** Parameters for variation of QOL between cities.

Parameter	Estimate	Std. Error	Z-value	*p*-value	Significance ^a^
**γ_1_**	0.792	0.548	1.44	0.14876	
**γ_2_**	−0.296	0.563	−0.53	0.59875	
**γ_3_**	3.135	0.578	5.43	5.72E-08	***
**γ_4_**	1.049	0.556	1.89	0.05903	*
**γ_5_**	0.443	0.560	0.79	0.4295	
**γ_6_**	−0.438	0.557	−0.79	0.43146	
**γ_7_**	−3.383	0.546	−6.2	5.62E-10	***
**γ_8_**	−0.900	0.528	−1.71	0.08807	*
**γ_9_**	−1.048	0.536	−1.95	0.05071	*
**γ_10_**	−0.625	0.560	−1.12	0.26434	
**γ_11_**	−0.200	0.567	−0.35	0.72457	

^a.^ * sig < 0.10, ** sig < 0.05, *** sig < 0.01.

The three systematic parameters measuring the impacts for (1) the city the respondent is from, (2) the city being evaluated, and (3) the interactive affect are all highly significant (Table 6). The first of these α1, described earlier as the cultural effect, indicates a significant positive shift in QOL ranks on responses from Bellingham panelists *versus* ones from Tateyama easily seen in each column of Figure 1. For example in looking at northwest quadrant *versus* the southwest quadrant (both in the left column) describing Tateyama, the black line clearly shifts down in the lower quadrant indicating Bellingham respondents systematically applied higher ranks to the questions then Tateyama. The same can be seen in the right hand column as well (northeast *versus* southeast). On the other hand looking across rows, same panel evaluating QOL indicators for the two different cities, Bellingham receives systematically more higher rankings which is also confirmed by the significantly positive α2 measure; or an indication of different resources and/or policies. Finally, for the interactive term δ, a significantly negative value appears indicating that Bellingham scores its own QOL lower than would be expected all else held constant. Again, this is interpreted as an indication of knowledge and experience attainable only through long term residency. Although not as easily spotted in Figure 1, a careful look at say level 1, the black lines, indicates that in the right hand column the distance between where Tateyama panelists and ones from Bellingham estimate this line is nearly equal, or a lower response (the black line moves up) than otherwise would be expected by Bellingham on itself. This means that Bellingham ranks itself lower than would otherwise be expected based on knowledge that we speculate is only available to long-time residents. Since this is a symmetrical relative measure and works for investigating either Tateyama or Bellingham, as second way to state this is that Tateyama scores itself higher than would be expected from all other measures. Based on this interpretation, a non-significant results for δ would indicate that long term residency in either city has no impact/offers no additional information.

**Table 6:** Systematic shift and interactive parameters.

Parameter	Estimate	Std. Error	Z-value	*p*-value	Significance ^a^
**α_1_**	1.943	0.458	4.25	2.18E-05	***
**α_2_**	2.010	0.391	5.14	2.73E-07	***
**δ**	−1.189	0.236	−5.04	4.61E-07	***

^a.^ * sig < 0.10, ** sig <0.05, *** sig < 0.01.

### 4.2. Testing the Model’s Assumptions and Fit

Three different methods were used to successfully demonstrate how well the model fits our assumptions. These are an F-test for goodness-of-fit, running a check on the proportional odds assumption, and a test of the random effect. Details are provided in an Appendix.

Two other means of evaluating the model’s reliability were used. The first is an ocular method, simply a visual assessment of how closely the expected logit lines in Figure 1 match the observed logit circles of similar color, which is a fairly tight throughout the figure although not a perfect fit. For example in the upper right hand corner (Tateyama Panel reporting on Bellingham) bins represented in increasing cumulative order, black, red, green, and purple quite closely track their appropriate lines, the highest cumulative bin light blue is not as tight, perhaps further indication of the more centrist responses of the Japanese participants. Other, graphs are similarly good fits.

Second is a more intuitive method of comparing the model’s parameter estimation with our earlier descriptive discussion of summary measures results. Although this is not a perfect method nor complete statistical analysis of our multivariate model *versus* simple descriptors, the closer the two the more confident we are of our results.

First, looking at the systematic shift parameters, α1, city of residency for a participant, and α2, city evaluated, both confirm our earlier conjecture that there is a significant cultural and resource/policy shift in responses. Tateyama panelists generally rank QOL indicators lower regardless of place evaluated and city of Bellingham is generally described as having higher QOL measures by both groups. Second, the model adds the interactive term (δ) which we have interpreted as a long term knowledge base. This measures additional information that is available only to residents of a city, not short time visitors, and it is not possible to capture using our earlier descriptive measures and tests.

Turning to the βk and γk parameters relating to the individual QOL indicators in our model it is beyond the scope of this short paper to completely explore their relationship to the average measures reported earlier in Table 2. However, as a quick overview the following can be noted.

For the βk parameters (Table 4) which directly impact each Tateyama QOL indicator, the first seven are all significant and a quick look at the signs indicates that the first 6 are predicted to be below the over-all QOL and the seventh one above. A review of Table 2 descriptives indicates that the mean values for Tateyama, regardless of the group reporting them, all follow this rule with the one exception. For indicator 2, the quality of transportation, the Bellingham panel scores this higher rather than lower. We find this not surprising since a review of the written responses of the Bellingham panelists contained many glowingly reports of mass transportation throughout Japan and Tokyo in particular and not merely inside Tateyama, where local residents stated it was not equally developed. The remaining four β’s are not significant, and again a review of Table 2 shows the means of these measures to be quite close to the over-all measure of QOL for Tateyama.

Turning to Bellingham, which is impacted by a combination of βk and γk coefficients, these results also tend to confirm the descriptive statistics. For example for the third indicator, parks and recreation, where each parameter is significantly different in value and sign the combined impact (β3 = −1.842 plus γ3 = 3.135) of the two parameters would be a bit positive (exact additive result of 1.293). In fact in reviewing Table 2, Tateyama (impacted only by β3) receives a value of 687 from Bellingham panelists and 470 from its own panelists, both of which are below Tateyama’s over-all estimates of QOL, 758 and 650 respectively, exactly what would be expected from β3 alone. But the combined parameters, as noted above, reverse the sign as do the results for Bellingham. The Bellingham panel reports 909 for parks and recreation for itself which is well above its over-all QOL estimate of 853, and the Tateyama panel indicates a similar result with 822 and 767 respectively. In a similar manner other results can also be reviewed.

From this we conclude that the model has a good fit with our data and hence that the latent structure identified by our model confirms the hypothesis that there is a general QOL framework upon which both Bellingham and Tateyama are located. This provides evidence for meeting the necessary conditions that cities can benefit from building international relationships and UNKN’s.

## 5. Conclusions

In the era of globalization urban networking is a growing trend turning former competitors into cooperators. Yet, as noted earlier, it is one thing for cities to network, but a completely different issue to derive value from such connections especially through social capital development and utilization by means of a UNKN. In nurturing such learning cities in pursuit of sustainability and high QOL, both necessary and sufficient conditions must be met in order for knowledge and value transfer and utilization to occur, particularly through cognitive social capital development. Using Proportional Odds Modeling with a Cumulative Logit Link to explore underlying latent structure for a two city SCTR professional exchange, this study has demonstrated that necessary conditions have been met—that there is a common QOL framework upon which each city falls despite their great cultural, resource, and policy differences. What remains for future work is demonstration that sufficient conditions are also met, that actual knowledge transfer occurs, and that value is realized from networking. Again, in prior studies this too was assumed to occur if indirect measures of structural social capital development were demonstrated. Thus, this study addresses one lacuna, the meeting of necessary conditions for cognitive social capital development from SCTRs, but leaves demonstration of meeting sufficient conditions as the next step. Other areas that also warrant additional exploration would be exploring relationships between cities of different sizes, cultures, and levels of economic development.

## Supplementary Files

Supplementary File 1

## Appendix

### A. Testing the Model’s Assumptions and Fit

**F-test goodness-of-fit:** the smallest possible model Logit[P(Yikm ≤ j)] = θj was compared to the full model (equation 1) based on the log-likelihood value from each, −1842.60 and −1533.586 respectively using the following X2 = 2(loglik(big-model) − loglik(small-model)) = 618.028 for 26 degrees of freedom (estimation of 31 parameters for big-model *versus* 5 for the small-model). The results show a significant difference between them where the *p*-value is zero to four decimal places and, hence, is well below 0.05. This result confirms the improvement using the proposed model.

**Checking the proportional odds assumption:** The proportional odds assumption (basically that the lines in Figure 1 should be parallel for each city) is a big assumption. This was tested by relaxing the assumption that there is a single β variable per question (that is allow a different β for each combination of question and rank j). Thus we compared the existing model of 11 β’s to a relaxed one with 55 β’s. The test comparing these models was performed via a likelihood ratio test with 44 degrees of freedom, because the models differ by 44 β parameters. The test statistic was 54.91846, giving a *p*-value of 0.12521. Hence, we conclude that the full model did not fit significantly better than the proportional odds model, or simply that our proportional odds assumption appears valid.

**Testing the random effect:** Recall that an individual’s random effect value is assumed to be a random draw from a normal distribution with a mean of zero and a variance of σ^2^. The value of σ^2^ is estimated in our model. To test if the model with random effects fits significantly better than the model without this effect, we just need to test whether σ^2^ (or, equivalently, σ) is different than zero. The estimated value of σ from this model was 1.403. A 95% profile likelihood confidence interval for σ was (1.121125, 1.791657). Since this does not contain zero, we conclude that σ is significantly different than zero (*p*-value less than 0.05).

## References

[1] Clarke N. (2009). In what sense ‘spaces of neoliberalism’? The new localism, the new politics of scale, and town twinning. Polit. Geogr..

[2] Clarke N. (2010). Town Twinning in Cold-War Britain: (Dis)continuities in Twentieth-Century Municipal Internationalism. Contemp. Br. Hist..

[3] Ogawa A. (2012). Sister City as Preservation Strategy. MS Theses.

[4] Zelinsky W. (1991). The twinning of the world: Sister Cities in geographic and historical perspective. Ann. Assoc. Am. Geogr..

[5] Bitsell B.M., Bulkeley H. (2006). Cities and the multilevel governance. Glob. Gov..

[6] Perkmann M. (2007). Policy entrepreneurship and multilevel governance: A comparative study of European cross-border regions. Environ. Plan. C.

[7] Blanco H., Campbell T. (2006). Social capital of cities: Emerging networks of horizontal assistance. Technol. Soc..

[8] Bouteligier S. (2013). Cities, Networks, and Global Environmental Governance: Spaces of Innovation, Places of Leadership.

[9] Sassen S. (2002). Global Networks, Linked Cities.

[10] Suzuki H., Dastur A., Moffatt S., Yabuki H. (2009). Eco2 Cities: Ecological Cities as Economic Cities, Conference Ed.. http://siteresources.worldbank.org/INTEASTASIAPACIFIC/Resources/226262–1246459314652/Eco2Cities_FrontMatter_ConfEdition6–26–09.pdf.

[11] Taylor P. (2004). World City Network: A Global Urban Analysis.

[12] UNDP (United Nations Development Program) The challenges of linking. Management Development and Governance Division, Bureau of Development Policy, New York, 2000. ftp://pogar.org/LocalUser/pogarp/other/undp/guides/challenglink-03e.pdf.

[B13-ijerph-12-07133] 13.For clarity and simplicity, Sister City Type Relationships will be used to designate all versions of Sister Cities’ relationships, twinning of cities, city-to-city agreements, *etc.* By such relationships, we mean those which extend across international boundaries and are freely entered into by two urban areas.

[14] Costanza R., Fisher B., Ali S., Beer C., Bond L., Boumans R., Danigelis N.L., Dickinson J., Elliott C., Farley J. An integrative approach to Quality of Life measurement, research, and policy. http://sapiens.revues.org/169.

[15] Campbell T. (2009). Learning cities: Knowledge, capacity and competitiveness. Habitat. Int..

[16] Campbell T. (2012). Beyond Smart Cities: How Cities Network, Learn, and Innovate.

[17] O’Dell C., Grayson C.J., Essaides N. (1998). If We Only Knew What We Know.

[18] Leresche J., Saez G., Perkmann M., Sum N. (2002). Political frontier regimes: Towards cross-border governance. Globalization, Regionalization and Cross-Border Regions.

[19] Campbell T. (2009). Torino as a Learning City.

[20] Buckley P., Belec J. (2011). Cascadia reconsidered: Questioning micro-scale cross-border integration in the Fraser Lowland. Univ. Fraser Valley Res. Rev..

[21] Aydalot P. (1986). Milieux innovateurs en Europe.

[22] SCI (Sister Cities International) Sister Cities International Statistics. http://www.sister-cities.org/.

[B23-ijerph-12-07133] 23.One must be cautious when using this metric since urban rank-size theory demonstrates a logarithmically increasing number of smaller cities as one moves down the ranks from a country’s primate city. Thus, although as an absolute count there are more cities under 100,000 participating in SCTRs that does not mean that they participate at proportionally a greater than expected rate. However, this is further complicated by the fact that larger cities tend to have many more SCTRs than smaller ones, for example Chicago IL has 28 while most smaller cities have only one or two.

[24] Hoetjes B. (2009). Trends and issues in municipal twinnings from the Netherlands. Habitat. Int..

[25] Cremer R., de Bruin A., Dupuis A. (2001). International sister cities: Bridging the global local divide. Am. J. Econ. Sociol..

[B26-ijerph-12-07133] 26.Note that this final goal represents a development beyond the bridging of understanding through “people to people” diplomacy championed by Eisenhower and others under the assumption that there was a common cognitive humanity that could be discovered through personal interaction. Technical staff exchanges are much more advanced and targeted and occur with the understanding that a common outlook on humanity has already been established. Instead, these exchanges open the question of whether there is a common cognitive outlook on issues of QOL and sustainability.

[27] Ishinabe N. (2010). Analysis of international city‐to‐city cooperation and intercity networks for Japanese national and local governments. http://enviroscope.iges.or.jp/modules/envirolib/upload/2789/attach/iges_discussion_paper_local_initiatives_march_2010.pdf.

[28] O’Toole K. (2000). From mates to markets: Perceptions of Australian sister city relationships. Policy Organisation Soc..

[29] O’Toole K. (2001). Kokusaika and internationalisation: Australian and Japanese sister city type relationships. Aust. J. Int. Aff..

[30] Ruthurford F. (2000). Sister cities report for the research and development scheme, December 2000. http://www.lga.sa.gov.au/webdata/resources/project/Sister_Cities_Report_-_LGA_2000.pdf.

[31] COB (City of Bellingham) Record of proceeding of city council city of Bellingham, Washington, Council Chambers, City Hall Monday, 3 October 1994, 07:00 PM Book:48, p. 1. http://www.cob.org/web/council.nsf/0/dc2009fb46b1d12388256107006100a2!OpenDocument&Click=.

[B32-ijerph-12-07133] 32.Unfortunately in the following year 2008 the deteriorating international financial situation and subsequent budget cuts put additional exchanges on hold, which has continued up to the present.

[B33-ijerph-12-07133] 33.The QOL indicators were only collected in the first round of a four round Delphi Study and might be thought of thought of as similar to the results from a single round questionnaire, although here they are used to frame the context within which the remaining rounds of the Delphi are conducted.

[B34-ijerph-12-07133] 34.Although we have developed this set of QOL indicators around three categories of the physical, service, and social environment of cities, reflecting more the way staff from a city would understand them, another way of understanding their development can be drawn from the work of Costanza *et al.* [14] (para. 3), who list categories under “basic needs for subsistence, reproduction, security, affection, *etc.*”. For example, physical indicators tend to align with subsistence, reproduction with social environment specifically provided to families and children, and security with services, *etc*.

[B35-ijerph-12-07133] 35.Note that significance has been placed at 0.10 a more liberal measure than is often times suggested, but given the small universe of potential respondents it is assumed justifiable.

[36] Lee J., Patricia W., Galbraith S., Jones P., Mineyama Y., Zhang X. (2002). Cultural differences in responses to a Likert scale. Res. Nurs. Health.

[37] Norris C., Ghali W., Galbraith P., Graham M., Jensen L., Knudtson M. (2004). Women with coronary artery disease report worse health-related quality of life outcomes compared to men. Health Qual. Life Out.

[38] Lall R., Campbell M., Walters S., Morgan K. (2002). A review of ordinal regression models applied on health-related quality of life assessments. Stat. Methods Med. Res..

[39] Lu M. (2002). Determinants of residential satisfaction: Ordered logit *vs.* regression models. Growth Change.

[40] Thakuriah P., Metaxatos P. (2000). Effect of residential location and access to transportation on employment opportunities. Transport Res. Rec..

[B41-ijerph-12-07133] 41.Our choice, and it was a choice, for using ordinal regression over linear was based on the following consideration. We felt that it is always best to choose the methodology that most correctly aligns with the data, which based on respondent preference was clearly more ordinal than interval. Further, linear regression assumes a randomness of error terms following a normal distribution. Our data does not look like this. If such randomness was true then we should not be getting large numbers of respondents with identical “y” values. Thus, we felt that it was more correct to choose a model that more accurately describes the behavior of the data and selected a type of ordinal regression.

[B42-ijerph-12-07133] 42.A single bin for answers 0 to 500 seemed reasonable since such a small portion of the results were below the 400 to 500 range. In fact the vast majority of all responses are included in this range, 213 out of 242; of these 176 had recorded values of exactly 500 and an additional four had a response of exactly 450. The next most common response was 400 with 33 responses. The remainders in this bin were as follows. No values below 100, three at 100, nine at 200, one at 250, 14 at 300, and two at 350.

[43] Agresti A. (2010). Analysis of Ordinal Categorical Data.

[44] Christensen R.H.B. (2015). Regression models for ordinal data R package Version 2015.1–21. http://www.cran.r-project.org/package=ordinal/.

[B45-ijerph-12-07133] 45.The observed logits are calculated based on the proportion of individuals who gave a score at-or-below a given number—which refers to our cumulative bin numbers in this study—divided by the proportion who gave a score above that number and then the ratio was logged and plotted.

