# Supplementary material for: The Role of Sister Cities’ Staff Exchanges in Developing “Learning Cities”: Exploring Necessary and Sufficient Conditions in Social Capital Development Utilizing Proportional Odds Modeling
Source: Int J Environ Res Public Health. 2015 Jun 24;12(7):7133–53. doi: 10.3390/ijerph120707133 (PMC4515646; doi:10.3390/ijerph120707133)
Supplement: Supplementary File 1 [file ijerph-12-07133-s001.zip › S2-Round 1 -- Questionnaire.docx]

**ROUND 1**

**Welcome** to **a Delphi Study of Sister Cities Exchanges and Quality of Life in Bellingham, WA and Tateyama, Japan.** Very briefly we will first define Quality of Life (QOL), a central theme of this study. Next we turn to the objective of this study and explain why we think that your opinions and thoughts are so important to us. Finally, we explain what a Delphi Study is, how it works, and provide the draft schedule for this particular study.

**Quality of Life (QOL)** is a feeling of wellbeing, satisfaction, and happiness that results from our personal relationship to factors in our world and environment, both the natural and the human. Hence the better the over-all environment in which we live and our ability to enjoy and benefit from it, the greater is our sense of fulfillment or QOL. Both Tateyama and Bellingham contain great natural and human environments and thus very high QOL. We believe that part of the reason they each has such a high QOL is because of their Sister City relationship (now almost 50 years old – one of the oldest between Japan and the USA!!!). This is because a Sister City relationship offers unique opportunities for building strong international friendships and also opportunities for learning from one another. Further, the better we learn from one another the better we can do things and hence improve our QOL, a simple but powerful thought.

**Study’s Objective:** The objective of this study is to identify the benefits of international exchanges between the Sister Cities of Tateyama and Bellingham that have occurred over the last several decades. Specifically, we wish to identify the most important impacts of these exchanges and learn how they bring us closer together as a world and help us to improve our local as well as global Quality of Life.

**Round One Questionnaire:** After some very short background questions in part one of this questionnaire, in part two to understand and measure QOL in each city we use *indicators of QOL* where we will ask you to evaluate and describe your satisfaction with things in each city. For example we will ask you to evaluate housing in both cities. We will also ask you how different this QOL indicator (housing) is in each place, since it is not completely comparable. Finally, in part three we ask you how the Sister City exchanges can impact Tateyama and Bellingham.

**Why your experiences and ideas are so important:** As a member of a Sister City exchange you have been in a unique position to experience a different culture from the inside and also to re-evaluate your own culture from afar before returning home. This makes you a very special person. Further, you have first hand knowledge about the impacts of these exchanges both in your own city and also in your Sister City. This knowledge is what this study seeks to discover. With your help and participation we hope to understand the benefits of a Sister City relationship and its impact on QOL.

**How the Delphi Study works:** A Delphi Study is a way to understand our complex world by exchanging information among a group of people called a panel based on a series of confidential written questionnaires organized into rounds. This is the first of four rounds.

However, a Delphi Study is much more than a simple questionnaire, since it involves first selecting a panel of knowledgeable people (in our case people that have participated in Sister City exchanges), and then asking them questions and then anonymously sharing the results among all panelists over a series of rounds. This makes it much more fun and valuable then a single questionnaire because the information grows during the rounds and each panelist can learn what others are thinking and add their own knowledge. However, like a questionnaire, the names of all participants remain confidential. All responses are collected by the researchers through regular mail or anonymous e-mail. Then only summaries of the results are shared back with the group. Thus, people express their opinions and learn of the opinions of others, but people’s names are never associated with responses. In fact not even the names of the panelists themselves are ever made public.

Another nice thing about a Delphi Study is that you can answer these questions at you leisure and since it is broken into 4 rounds, no one round should take you more then 15 or 20 minutes to complete. These rounds will occur over a couple of months, so that it is quite easy to find time to respond.

Finally this study is unique! In the last round, round four, information provided by both panelists in Tateyama and Bellingham will be anonymously shared. (Results from Bellingham will be translated into Japanese and results from Tateyama will be translated into English). So not only will panelists learn about their own experiences but also the benefits that their Sister City has experienced.

| **Draft Schedule** | |
| --- | --- |
| **Early July** |  |
| **Week 1** | Send Round One Questionnaire |
|  |  |
| **Week 2** | Receive and Analyze Reponses |
|  |  |
| **Week 3** | Send Round Two Questionnaire |
|  |  |
| **Week 3** | Receive and Analyze Reponses |
|  |  |
| **Week 4** | Send Round Three Questionnaire |
|  |  |
| **August** | Translate both Tateyama and Bellingham Responses from Round Three |
|  |  |
| **September** | Send Round Four to people in both Tateyama and Bellingham |
|  |  |
| **November** | Share Final Report with both cities |

**Are you ready? Let’s begin, please go to the next page and please send your response with one week.**

**Part 1: General Background**

1. How many times have you visited the USA? (Circle the correct answer)

**1 2 3 4 5 more than 5**

2. How many times have you visited Bellingham? (Circle the correct answer)

**1 2 3 4 5 more than 5**

**First Exchange Visit to Bellingham**

1. Approximately when was your first exchange visit to Bellingham?

(Check one box)

- **Within the last 5 years (between 2001 and 2006)**
- **Between 5 and 10 years ago (between 1995 and 2000)**
- **More than 10 years ago (before 1995)**

1. Approximately how many weeks did you stay in Bellingham?
   - **1 week or less**
   - **2 weeks**
   - **3 weeks**
   - **4 weeks**
   - **Other please specify ___________________________________________________**

5. For your first visit to Bellingham what kind of a visit was it? (Check all that apply)

- **City Hall Staff Exchange**
- **Educational Exchange**
- **Friendship/Tourism Exchange such as “Ski to Sea”**
- **Other kind -- please describe _________________________________________**

6. Why did you want to visit Bellingham? (Check all that apply)

- **Professional development**
- **Educational opportunity**
- **Cultural exchange and make new friends**
- **Practice English**
- **Other reasons -- please describe _________________________________________**

7. Before your visit to Bellingham how much did you know about Bellingham?

(Please choose a number on the following scale)

**Knowledge of Bellingham before visit**

**1 2 3 4 5 6 7 8 9 10**

Little some quite a bit a lot to none

8. When you arrived in Bellingham was it different or the same as you expected?

**1 2 3 4 5 6 7 8 9 10**

As expected a little different a lot different very different

Finally

9. Gender

- **Male**
- **Female**

10. Current Age

- - **19 years old or less**
  - **20 – 29 years old**
  - **30 - 39**
  - **40 – 49**
  - **50 - 59**
  - **60 - 69**
  - **Over 69**

**Part 2: Evaluation of Quality of Life Indicators**

In this section you will be asked to think about indicators that affect the Quality of Life in each of the Sister Cities and then respond to three short questions and make comments about each indicator:

1. **Quality of the indicator in Bellingham:** Second on a scale of 1 to 1,000 indicate how good the indicator is in Bellingham, where 1,000 is perfect. **Example:** Again, for Food it might have been very good, but perhaps not as good as in Seattle or New York City, so you might score that as 940.
2. **Quality of the indicator in Tateyama:** Third scale the same indicator again on a scale of 1 to 1,000 but this time based on your experience in Tateyama. **Example:** Again for food, you might decide that it is quite good in Tateyama, but not perfect and not as good as in nearby Tokyo. So you might score food as 825 in Tateyama.

Following are the QOL Indicators, please score them based on your visit to Bellingham and your experience in Tateyama,

**Q1--Housing**

**How good is the quality of Housing in each city with 1,000 being perfect?**

**A.** Bellingham (enter a score of 1 to 1,000) _______________

**B.** Tateyama (enter a score of 1 to 1,000) _______________

**Q2--Transportation**

**How good is the quality of Transportation in each city with 1,000 being perfect?**

**A.** Bellingham (enter a score of 1 to 1,000) _______________

**B.** Tateyama (enter a score of 1 to 1,000) _______________

**Q3--Parks and Outdoor Recreation Opportunities**

**How good are the Parks and Outdoor Recreation Opportunities in each city?**

**A.** Bellingham (enter a score of 1 to 1,000) _______________

**B.** Tateyama (enter a score of 1 to 1,000) _______________

**Q4--Opportunities for Leisure and Amusement Activities**

**How good are the Opportunities for Leisure and Amusement in each city?**

**A.** Bellingham (enter a score of 1 to 1,000) _______________

**B.** Tateyama (enter a score of 1 to 1,000) _______________

**Q5--Opportunities for Educational Enrichment**

**How good are the Opportunities for Educational Enrichment in each city with 1,000 being perfect?**

**A.** Bellingham (enter a score of 1 to 1,000) _______________

**B.** Tateyama (enter a score of 1 to 1,000) _______________

**Q6--Opportunities for Shopping**

**How good are the Opportunities for Shopping in each city with 1,000 being perfect?**

**A.** Bellingham (enter a score of 1 to 1,000) _______________

**B.** Tateyama (enter a score of 1 to 1,000) _______________

**Q7--Personal Safety and Security**

**How good is a feeling of Personal Safety and Security in each city with 1,000 being perfect?**

**A.** Bellingham (enter a score of 1 to 1,000) _______________

**B.** Tateyama (enter a score of 1 to 1,000) _______________

**Q8--Environment for Families**

**How good is the Environment for Families in each city with 1,000 being perfect?**

**A.** Bellingham (enter a score of 1 to 1,000) _______________

**B.** Tateyama (enter a score of 1 to 1,000) _______________

**Q9--Environment for Children**

**How good is the Environment for Children in each city with 1,000 being perfect?**

**A.** Bellingham (enter a score of 1 to 1,000) _______________

**B.** Tateyama (enter a score of 1 to 1,000) _______________

**Q10--Environment for Retired People**

**How good is the Environment for Retired People in each city with 1,000 being perfect?**

**A.** Bellingham (enter a score of 1 to 1,000) _______________

**B.** Tateyama (enter a score of 1 to 1,000) _______________

**Q11--Feeling of Community and Volunteer Spirit**

**How good is the Feeling of Community and Volunteer Spirit in each city with 1,000 being perfect?**

**A.** Bellingham (enter a score of 1 to 1,000) _______________

**B.** Tateyama (enter a score of 1 to 1,000) _______________

**Q12--Over-all Quality of Life**

**How good is the Over-all Quality of Life in each city with 1,000 being perfect?**

**A.** Bellingham (enter a score of 1 to 1,000) _______________

**B.** Tateyama (enter a score of 1 to 1,000) _______________
